# Foregut Anguish: A Rare Case of Median Arcuate Ligament Syndrome in Common Variable Immunodeficiency

**DOI:** 10.7759/cureus.4865

**Published:** 2019-06-10

**Authors:** Syed Hamza Bin Waqar, Aiman Rehan, Qirat Jawed, Hasham Saeed, Khizer Siddiqui

**Affiliations:** 1 Internal Medicine, Civil Hospital Karachi, Karachi, PAK; 2 Internal Medicine, Dow University of Health Sciences, Karachi, PAK; 3 Internal Medicine, Dow Medical College and Civil Hospital, Karachi, PAK; 4 Miscellaneous, Dow Medical College, Karachi, PAK

**Keywords:** cacs, mals, median arcuate ligament, vascular stenosis, dunbar syndrome, mesenteric ischemia, postprandial pain, celiac artery stenosis

## Abstract

Median arcuate ligament syndrome (MALS), also known as celiac artery compression syndrome (CACS), is a rare clinical entity that is characterized by extrinsic compression of the celiac artery by the median arcuate ligament of the diaphragm. It mostly presents as a triad of post-prandial abdominal pain, weight loss, and bruit in the epigastrium. Given its variable and atypical presentation, it is a diagnostic challenge for physicians. MALS is supposed to be a diagnosis of exclusion and, thus, many investigations need to be done before coining it as a definitive diagnosis. Herein, we present a case of a 17-year-old female patient, a known case of common variable immunodeficiency (CVID) who presented to our setup with progressive, excruciating epigastric pain and bilious vomiting after undergoing excision of CVID-associated gastric adenocarcinoma (CAGA). Multiple sets of tests were conducted to rule out possible cardiopulmonary, musculoskeletal, and abdominal etiology. The diagnosis was made on the basis of findings depicted on three-dimensional computed tomographic angiography (3D-CTA) and ultrasound Doppler studies of the celiac artery. She underwent laparoscopic dissection of the median arcuate ligament with a celiac plexus block, which yielded immediate relief in symptomatology and was later followed up with duplex scans and showed complete remission of symptoms.

## Introduction

The phenomenon of ischemic abdominal pain caused by the median arcuate ligament extrinsically compressing the celiac artery has been known since 1917 [1]. However, the pathophysiology behind the cascade of events that lead to median arcuate ligament syndrome (MALS), also known as celiac artery compression syndrome (CACS), is still uncertain to date. Ligamentous compression of the celiac trunk can be asymptomatic in around 10% to 24% of patients, with only radiographic evidence of compression. Thus, it has been postulated that some degree of neural compression of the adjacent celiac plexus via fibrous and periaortic tissue is also a part of the pathogenesis [2]. The complexity of clinical presentation and the radiological paradigm makes the diagnosis of CACS a dilemma for physicians. Herein, we report the first case, to our knowledge, of MALS in a young patient of 17 years diagnosed with common variable immunodeficiency (CVID). She developed this condition after undergoing resection of her CVID associated gastric adenocarcinoma (CAGA).

## Case presentation

A 17-year-old female, a diagnosed case of common variable immunodeficiency (CVID) for six years, presented to our tertiary set-up with progressive abdominal pain and vomiting for seven weeks. The pain had started gradually in the epigastrium, two days after undergoing radical gastrectomy for a diagnosed multifocal intestinal-type adenocarcinoma. It was first noted as mere discomfort in the abdomen but in a few days, it progressed in intensity. It was non-radiating, sharp, and gripping in nature, and more localized to the lower chest and epigastric region. It aggravated peculiarly after intake of food or liquids and was relieved on fasting or leaning forward. Mild pain was always present in the background and intensified whenever she ate anything, to the point that she wasn’t able to tolerate any solids or liquids. The pain was associated with frequent episodes of non-projectile bilious vomiting, about seven to eight times a day and accompanied by nausea. She also lost around 26 pounds of weight in seven weeks. These episodes of pain caused depravity in her quality of life and limitations in her activities of daily living (ADL). She also felt fatigued. There was no associated fever, night sweats, bloating, post-prandial fullness, diarrhea, constipation, dysphagia, or melena. She had a past medical history of pan-hypogammaglobulinemia, pernicious anemia, recurrent sinusitis and pneumonia, and two hospitalizations for complicated cases of pneumonia. Past surgical history was significant for a subtotal radical gastrectomy, which was performed for early stage CVID-associated gastric adenocarcinoma (CAGA) involving the antrum and pyloric region but not extending beyond submucosa.

On admission, she looked frail, wasted, and in significant discomfort but was alert and oriented, with no mood alterations. The patient was afebrile, with a pulse of 98 beats per minute (BPM), blood pressure of 90/74 mmHg, and a respiratory rate of 14 per minute. Her abdomen was non-distended, slightly tender to deep palpation in the epigastrium, with normoactive bowel sounds in all four quadrants and no signs of hepatosplenomegaly. There was no bruit auscultated in the epigastrium on either inhalation or exhalation. The patient had no lymphadenopathy, edema, cyanosis, clubbing, conjunctival pallor or discharge, jaundice, rashes, oral ulcers, or thrush. The cardiovascular, respiratory, and nervous systems were normal on physical examination.

The complete blood count (CBC) showed a total leukocyte count (TLC) of 4.03 x 109/L, with neutrophils being 84% and lymphocytes being 10%. Platelets were 356 x 109/L. Mean corpuscular volume (MCV) was marginally elevated. Erythrocyte sedimentation rate and C - reactive protein levels were within normal limits. The coagulation profile was normal as well. The electrolyte panel indicated a sodium level of 129 mEq/L (normal: 136-146mEq/L) and potassium of 3.7 mEq/L (normal: 3.5-5.0mEq/L), consistent with the volume status. Urine urea levels were 25 mg/dl (normal: 6-20 mg/dl). The glycosylated hemoglobin (HbA1c) level was 4.3%, with a random blood sugar level of 81 mg/dl (normal: 79-160mg/dl). Amylase and lipase levels were obtained, which were in the normal range, ruling out pancreatitis. Liver function tests (LFTs) were also normal, ruling out hepatic cytolysis observed in post-surgical cases of celiac artery compression syndrome. Serum immunoglobulin G (IgG) was undetectable and serum immunoglobulin A and M were subnormal. Electrocardiogram (EKG), chest X-ray (CXR), and echocardiography (ECHO) were also done but turned out to be negative, ruling out the cardiopulmonary causes of pain. Furthermore, the lipid panel was insignificant.

Imaging and invasive testing were conducted. Esophagogastroduodenoscopy (EGD) was consistent with postoperative changes showing no alteration of the mucosa. Ultrasound (US) abdomen and computed tomography (CT) abdomen were also found to be normal.

Three-dimensional computed tomographic angiography (3D-CTA) was performed to visualize the intra-luminal and extra-luminal causes of compression of the celiac artery. It highlighted a focal non-atherosclerotic narrowing of the proximal celiac artery with a characteristic hooked appearance, suggestive of compression by the median arcuate ligament. There was some post-stenotic dilatation, but no significant collateralization or pseudo-aneurysmal changes in the pancreaticoduodenal artery were noted.

US duplex was also obtained, which showed elevated peak systolic (PS) and end-diastolic (ED) velocities at the origin of the celiac artery indicating stenosis. It particularly increased on expiration and decreased on inspiration.

The patient was then subjected to interventional radiology for a celiac plexus block (CPB); after which, she obtained relief in her pain and vomiting. The decision was made to re-explore the abdomen laparoscopically and the median arcuate ligament was dissected with the release of the celiac artery. Intra-operative Doppler sonography was also obtained to note the indices after median arcuate ligament release. The patient was later followed over a period of six months. She had no recurrence of symptoms and the parameters on the duplex US also remained within normal limits.

## Discussion

Celiac artery compression syndrome (CACS) is a rare vascular disorder defined as recurrent abdominal pain that is associated with the extrinsic compression of the celiac artery by the median arcuate ligament. It is characterized mostly by a triad of post-prandial abdominal pain, weight loss, and an occasional abdominal bruit. The diagnosis of CACS or median arcuate ligament syndrome (MALS) is often one of exclusion, given the deceptive and nonspecific symptoms that overlap with variants of intestinal ischemia. It is known to affect females four times more as compared to males, with age mostly ranging between 20 to 50 years. It strikingly affects females with a thin and lean habitus [2]. Its anatomical intricacy was first coined by Lipshutz B, in 1917, but the first reported case series that particularly talked about the management of this condition was published in 1965 by the radiologist JD Dunbar [3].

The pathophysiology of MALS remains uncertain, and many theories have been devised to explain the treatment and diagnostic aspects of this disease. Celiac artery compression can be noted in 10%-24% of patients without ischemic symptoms and their radiological imaging might show celiac artery stenosis up to 50%, making it the commonest single vessel stenosis in the abdomen. This has led to the belief that its pathogenesis is multifactorial in origin, making celiac plexus and periaortic ganglionic tissue an important part of MALS [4-5]. The median arcuate ligament is a fibrous band connecting the right and left diaphragmatic crura across the aortic hiatus at the level of the T12/L1 vertebral bodies. A low insertion of the ligament or a higher origin of the celiac artery can cause significant stenosis leading to hemodynamic instability and formation of collaterals between the celiac artery and the superior mesenteric artery for hemodynamic compensation. As the blood flow demand increases, it is diverted from the superior mesenteric artery to the celiac collaterals. This explains the post-prandial and exercise-related mesenteric pain observed in MALS [6]. The celiac ganglion and plexus sit adjacent to the ligament that originates from the preganglionic splanchnic nerves, somatic branches from the phrenic and vagus nerves, parasympathetic preganglionic nerves, and sympathetic postganglionic fibers. These nerves may also contribute to celiac artery compression [7]. Genetic causes and delayed gastric emptying can also work as causative agents for MALS [8-9].

The spectrum of presentation in MALS is best studied when divided into the two variants of presentation, namely, Dunbar and Sutton syndrome, respectively [10]. Dunbar syndrome is characterized by postprandial pain attributed to reduced blood flow resulting from functional celiac stenosis caused by the compression of the median arcuate ligament. It can also cause vomiting, weight loss, nausea, delayed gastric emptying, anorexia, and diarrhea. Stenosis of the celiac artery can reverse the blood flow in the gastroduodenal artery so that the liver and spleen are now supplied by the pancreaticoduodenal arcades and superior mesenteric artery. This unphysiological flow leads to shear stress in the pancreaticoduodenal artery’s (PDA) vascular wall, resulting in the formation of true and pseudo-aneurysms. This is referred to as Sutton syndrome and can often present as abdominal pain or retroperitoneal or chronic gastrointestinal hemorrhage and can be life-threatening [10-11]. Such aneurysms, owing to their weak vasculature, should be subjected to treatment. Aneurysms, even as small as 2 mm, have been known to rupture. This further emphasizes the importance of timely management of all aneurysms, irrespective of size [12]. MALS has been documented in patients with procedures like gastric bypass and pancreaticoduodenectomy [13-14].

The diagnosis of MALS is one of exclusion, mandating various tests to be done. Before the advent of modern imaging modalities, conventional angiography was used to make a diagnosis of MALS. It showed indentation on the celiac artery. Compression of the celiac artery is a dynamic process dependent on the phase of respiration, compression being less on inspiration, as the celiac axis assumes a more caudal orientation as the lung expands than on expiration when the aorta and celiac artery moves cranially. It is to be noted that isolated compression of the celiac axis in expiration may not be clinically significant and, hence, a correlation between the history and hemodynamic alteration should be accounted as the guiding principle in the diagnosis and management of MALS [15-16].

The thin-slice multidetector three-dimensional computed tomographic angiography (3D-CTA) and Doppler ultrasound studies of the celiac artery are increasingly being employed as non-invasive diagnostic tests and have replaced the previously used traditional catheter-based angiography. To comment on MALS, CTA should be obtained in both deep inspiration and deep expiration so that the changes in the diameter of the celiac artery in phases of respiration can be duly noted. Focal non-atherosclerotic narrowing of the proximal celiac artery, more pronounced in end-expiration, is characteristic of MALS with a typical hooked appearance of the narrowed segment. To be clinically reasonable enough to cause ischemia, stenosis should be above 70% of the caliber. Post-stenotic dilatation may be visible along with a massive network of collaterals between the branches of the celiac artery and superior mesenteric artery, especially via the pancreaticoduodenal arcade. Aneurysms, along with the pancreaticoduodenal artery and dorsal pancreatic artery, can also be visualized. Duplex ultrasound is also performed as an adjunct to CTA. Elevated systolic velocities and end-diastolic velocities, especially in expiration, are pertinent with the diagnosis of MALS. In one review, the duplex ultrasound revealed a sensitivity and specificity of 83% and 100%, respectively. Parameters of a peak systolic velocity of above 350 cm/second, a 210% change in pulse volume amplitude with inspiration and expiration, and a deflection angle of 50 degrees were noted to be reliable for diagnosis [15-18].

Patients with history, imaging, and hemodynamic parameters consistent with MALS should be referred to laparoscopic skilled surgeons, anesthesiologists, and interventional radiologists. A joint venture of a celiac plexus block with lidocaine/bupivacaine or ganglionectomy with median arcuate ligament release has known to cause documented remission in MALS. If employed, a laparoscopic approach has shown to have a shorter hospital stay, faster recovery, decreased blood loss, and a better cosmetic outcome as compared to the open approach. Fujiwara Y et al. suggested that the diaphragmatic fibers should be incised for approximately five centimeters in a cephalad direction, exposing up to 4 cm of the aorta. Intraoperative assessment of the celiac artery flow can also be utilized either via direct vision or by duplex to ascertain the need for further decompression [6,19].

## Conclusions

Median arcuate ligament syndrome (MALS) poses a great diagnostic challenge due to its ambiguous and infrequent occurrence. This is a unique case of MALS in a patient with CVID, manifesting after the resection of a tumor. It is important to note that patients with post-prandial pain should be evaluated for vascular causes after all causes of mesenteric ischemia have been exhausted. Celiac plexus block (CPB) and laparoscopic release of the median arcuate ligament serve as a better treatment modality for MALS as compared to the open procedures. The literature on this entity is scarce and, hence, more studies should be conducted to probe further into the pathophysiology and management approaches that can be used to devise a definitive algorithm.
